# KRAS G12C–Targeted Therapy in Non-Small Cell Lung Cancer: From Resistant Salvage to Potential First-Line Backbone

**DOI:** 10.3390/ijms27146455

**Published:** 2026-07-20

**Authors:** Daniel Rosas, Priyanka Barad, Jervon Wright, Luis Raez

**Affiliations:** 1 Memorial Cancer Institute, Hematology Oncology Fellowship, Hollywood, FL 33021, USA; jwright@mhs.net (J.W.); lraez@mhs.net (L.R.); 2 Harnett Health Internal Medicine Residency, Dunn, NC 28334, USA; pbarad@mhs.net

**Keywords:** KRAS G12C, non-small cell lung cancer, sotorasib, adagrasib, targeted therapy, drug resistance, STK11, KEAP1, tumor microenvironment, precision oncology

## Abstract

KRAS G12C, long considered an undruggable oncogenic driver, has become one of the most consequential therapeutic targets in non-small cell lung cancer (NSCLC). The discovery of a cryptic binding pocket accessible in the GDP-bound state enabled covalent inhibitors—sotorasib and adagrasib—that have received regulatory approval for previously treated KRAS G12C-mutant NSCLC, with sotorasib demonstrating PFS and OS superiority over docetaxel in CodeBreaK 200 and adagrasib showing meaningful intracranial activity and a progression-free survival benefit over docetaxel in KRYSTAL-12. Yet response durability is limited by on-target switch-II pocket mutations, upstream RTK and SHP2-mediated bypass signaling, downstream MAPK and PI3K-AKT reactivation, phenotypic plasticity, and adverse modulation by co-occurring STK11, KEAP1, and TP53 alterations. Next-generation covalent inhibitors (divarasib, glecirasib, olomorasib), tri-complex RAS(ON) inhibitors (RMC-6291), pan-KRAS agents, and rationally designed combinations with EGFR, SHP2, SOS1, and PD-1 inhibitors are repositioning KRAS-directed therapy toward earlier lines of treatment. This review integrates the structural, signaling, and clinical biology of KRAS G12C with contemporary trial and real-world evidence to examine the emerging case for first-line KRAS G12C inhibition in genomically defined subsets of NSCLC. First-line use nonetheless remains investigational; platinum-based chemoimmunotherapy remains the standard of care outside of clinical trials, and a frontline indication will require confirmation from randomized phase III trials.

## 1. Introduction

Non-small cell lung cancer (NSCLC) accounts for more than 80% of lung cancer diagnoses and remains the leading cause of cancer-related mortality worldwide. Despite advances in screening, immunotherapy, and targeted agents, the prognosis for advanced NSCLC remains poor, with five-year survival rates below 20%. The advent of molecular stratification has transformed the therapeutic landscape, enabling precision medicine approaches that target specific oncogenic drivers. Among these, mutations in the Kirsten rat sarcoma viral oncogene homolog (KRAS) are the most prevalent, occurring in approximately 25–30% of NSCLC cases, particularly in adenocarcinoma and in patients with a history of smoking.

KRAS mutations are heterogeneous, with the G12C variant representing roughly 40–45% of KRAS-mutant NSCLC. Other variants such as G12D, G12V, G12S, and Q61 differ in their biochemical properties and therapeutic vulnerabilities. The G12C substitution introduces a reactive cysteine residue at codon 12, distinguishing it structurally and functionally from other KRAS mutants. This unique feature has enabled the development of covalent inhibitors that selectively bind to the mutant cysteine in its inactive GDP-bound state.

For decades, KRAS was considered “undruggable” due to its picomolar affinity for GTP/GDP and the absence of suitable binding pockets. Numerous attempts to target KRAS directly or indirectly through downstream effectors yielded limited clinical success. The paradigm shifted in 2013 when Ostrem et al. demonstrated that the switch-II pocket (S-IIP) in GDP-bound KRAS G12C could be selectively targeted, enabling mutant-specific inhibition [1]. This breakthrough enabled the rational design of covalent inhibitors such as sotorasib and adagrasib, which irreversibly bind to the mutant cysteine, locking KRAS in its inactive state and attenuating downstream signaling [2,3,4].

Initially developed as salvage therapies for heavily pretreated patients, KRAS G12C inhibitors have demonstrated meaningful clinical activity, prompting accelerated regulatory approvals [2,4]. More recently, combination strategies with immune checkpoint inhibitors and chemotherapy have expanded their therapeutic potential, positioning G12C inhibitors as legitimate contenders for first-line treatment [5]. This evolution reflects a broader shift in oncology: from empirical cytotoxic regimens to precision-guided, biomarker-driven interventions.

Given the biochemical specificity of G12C targeting, the molecular signaling consequences of KRAS inhibition, and the translational trajectory from bench to bedside, a comprehensive review is warranted. By examining the molecular underpinnings and clinical implications of KRAS G12C inhibition, this review contextualizes its emergence within the broader framework of NSCLC management and precision oncology.

### Review Scope and Search Strategy

This article is a narrative review rather than a systematic review. Relevant English-language literature was identified through PubMed/MEDLINE searches combining the terms “KRAS G12C,” “sotorasib,” “adagrasib,” “non-small cell lung cancer,” “acquired resistance,” and “combination therapy,” supplemented by hand-searching of reference lists and by abstracts and presentations from the ASCO, ESMO, AACR, and WCLC annual meetings through June 2026. Priority was given to pivotal clinical trials, mechanistic studies, and regulatory decisions relevant to the biology, resistance, and therapeutic sequencing of KRAS G12C-mutant NSCLC. Because study selection was guided by author judgment rather than predefined inclusion and exclusion criteria, a formal risk-of-bias assessment and quantitative synthesis were not performed, and selection bias cannot be excluded; the evidence is therefore summarized qualitatively and weighted toward randomized and prospective data where available.

## 2. Biology of KRAS and the G12C Oncogenic Driver

### 2.1. KRAS Structure, Function, and Nucleotide Cycling

KRAS belongs to the RAS family of small GTPases, which function as molecular switches regulating cell proliferation, differentiation, and survival. Structurally, KRAS consists of a highly conserved G-domain responsible for nucleotide binding and hydrolysis, and a hypervariable region that mediates membrane localization. Its activity is determined by cycling between an active GTP-bound state and an inactive GDP-bound state.

The transition between active and inactive states is tightly controlled by guanine nucleotide exchange factors (GEFs) and GTPase-activating proteins (GAPs). GEFs such as SOS1 facilitate the release of GDP, allowing GTP binding and activation. Conversely, GAPs such as NF1 accelerate GTP hydrolysis, returning KRAS to its inactive state. Mutations at codon 12, 13, or 61 impair intrinsic GTPase activity or GAP-mediated hydrolysis, leading to constitutive activation and persistent downstream signaling [6].

### 2.2. Downstream Signaling Pathways

**Figure 1:**
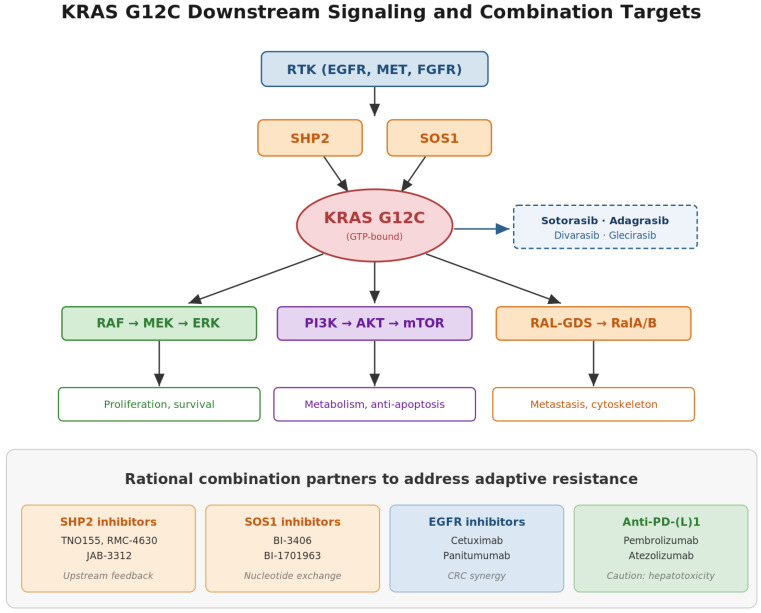
KRAS G12C downstream signaling pathways and rational combination targets. RTKs activate RAS through SHP2 and SOS1. GTP-bound KRAS drives the RAF-MEK-ERK cascade (proliferation), PI3K-AKT-mTOR pathway (survival), and RAL-GDS pathway (cytoskeleton/metastasis). Four classes of combination partners are shown: SHP2 inhibitors, SOS1 inhibitors, EGFR antibodies (cetuximab/panitumumab), and anti-PD-(L)1 agents.

Activated KRAS engages multiple effector pathways: the RAF-MEK-ERK cascade, which promotes cell proliferation and differentiation; the PI3K-AKT-mTOR pathway, which regulates survival, metabolism, and resistance to apoptosis; and the RAL-GDS pathway, which influences cytoskeletal dynamics, vesicle trafficking, and metastasis. The convergence of these pathways underlies the oncogenic potency of KRAS mutations, driving tumor initiation and progression [7,8] (Figure 1).

### 2.3. Unique Biology of KRAS G12C

**Figure 2:**
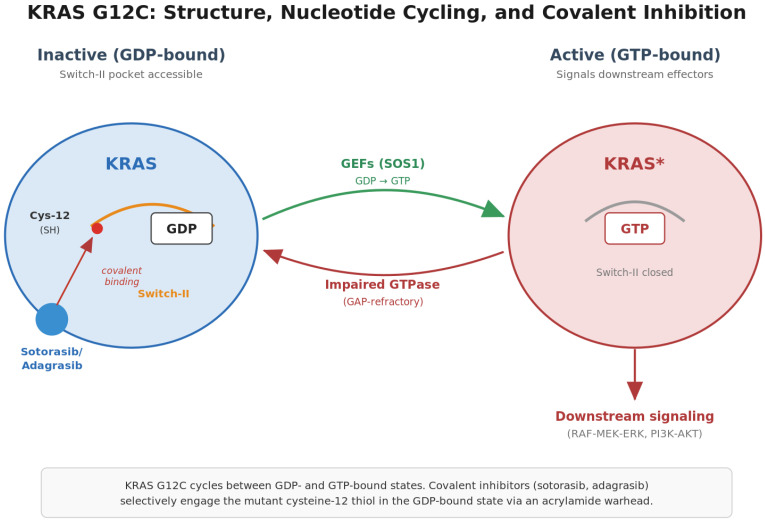
Structural basis of KRAS G12C covalent inhibition. KRAS G12C cycles between an inactive GDP-bound state, in which the switch-II pocket is accessible, and an active GTP-bound state (KRAS*), which engages downstream effectors. GEFs such as SOS1 drive GDP-to-GTP exchange; impaired GTPase activity (GAP-refractory) slows return to the inactive state. Sotorasib and adagrasib selectively react with the mutant Cys-12 thiol in the GDP-bound state via an acrylamide warhead, trapping KRAS G12C in the inactive conformation.

The G12C mutation substitutes glycine with cysteine at codon 12, introducing a nucleophilic thiol group. This biochemical feature enables covalent binding by small molecules designed to exploit a transient pocket in the inactive GDP-bound conformation [1,3]. Unlike other KRAS mutants, G12C retains partial cycling between active and inactive states, allowing therapeutic interception during its GDP-bound phase. This property is critical, as it permits selective inhibition without broadly affecting wild-type KRAS or other isoforms [1,3] (Figure 2).

KRAS G12C exhibits impaired GTP hydrolysis but does not remain exclusively in the active state. A sub-population of the protein resides in the GDP-bound conformation at any given time, providing a therapeutic window for covalent inhibitors. This dynamic cycling distinguishes G12C from variants such as G12D or G12V, which are more persistently active and lack druggable cysteine residues [9,10].

### 2.4. Spatial Heterogeneity and Co-Occurring Alterations

KRAS-mutant NSCLC is characterized by spatial heterogeneity and frequent co-occurring genomic alterations. Mutations in STK11 and KEAP1 are common, often conferring resistance to immunotherapy and influencing metabolic reprogramming [11,12]. TP53 co-mutations are also prevalent, shaping tumor biology and therapeutic response. These co-alterations underscore the complexity of KRAS-driven oncogenesis and highlight the need for combination strategies that address parallel pathways [13].

### 2.5. Diagnostic Considerations

Accurate detection of KRAS mutations is essential for patient stratification. Tissue-based next-generation sequencing (NGS) remains the gold standard, offering comprehensive genomic profiling [14]. However, liquid biopsy approaches using circulating tumor DNA (ctDNA) provide a minimally invasive alternative, enabling dynamic monitoring of mutational status and resistance mechanisms. Distinguishing KRAS G12C from non-G12C variants is critical, as only G12C harbors the reactive cysteine amenable to covalent inhibition. Misclassification may lead to inappropriate therapy selection and suboptimal outcomes [15].

## 3. Therapeutic Landscape of KRAS G12C Targeting in 2026

### 3.1. Sotorasib

Sotorasib (AMG 510) is a first-in-class small molecule that irreversibly binds the mutant cysteine of KRAS G12C in its GDP-bound inactive state, locking the protein in an inactive conformation and preventing downstream RAS-MAPK and PI3K-AKT signaling, thereby inducing tumor cell apoptosis and growth arrest [2]. Its efficacy depends on residual GTPase activity maintaining a GDP-bound pool. In colorectal cancer, KRAS G12C inhibition alone induces MAPK pathway rebound via EGFR-mediated feedback, limiting the efficacy of single-agent therapy; these findings support vertical co-inhibition strategies combining KRAS G12C and EGFR inhibitors [16,17]. Additionally, sotorasib cannot inhibit the GTP-bound active KRAS pool, and its CNS penetrance is limited by high molecular weight and P-glycoprotein susceptibility [2,3]. To address these pharmacologic constraints, combination approaches are being explored clinically, including SHP2 inhibitors to block RTK rebound, SOS1 inhibitors to regulate KRAS nucleotide cycling, and EGFR-directed therapies to prevent adaptive resistance [6,16,18].

In the phase I CodeBreaK 100 trial, sotorasib demonstrated an objective response rate (ORR) of 32.2% and a disease control rate (DCR) of 88.1% in patients with previously treated KRAS G12C-mutant NSCLC, with Grade ≥3 treatment-related adverse events (TRAEs) observed in 11.8% of participants [2]. In the phase II expansion cohort (*n* = 126), sotorasib achieved an ORR of 37.1%, a DCR of 80.6%, a median duration of response (DoR) of 11.1 months, a median progression-free survival (PFS) of 6.8 months, and a median overall survival (OS) of 12.5 months, with hepatotoxicity and diarrhea representing the most frequent clinically significant adverse events [2]. In the phase III CodeBreaK 200 trial, sotorasib demonstrated superior efficacy over docetaxel in patients with previously treated KRAS G12C-mutant NSCLC, with an ORR of 28.1% versus 13.2% and a PFS of 5.6 versus 4.5 months (hazard ratio [HR] 0.66; *p* = 0.002), although no OS benefit was observed (10.6 vs. 11.3 months; HR 1.01), likely due to crossover. On the basis of these confirmatory data, the manufacturer sought conversion of sotorasib’s accelerated approval to regular approval; however, following an October 2023 ODAC review that questioned the interpretability of the CodeBreaK 200 PFS result, the FDA issued a Complete Response Letter in December 2023 and declined regular approval. Sotorasib therefore remains under accelerated approval for NSCLC in the United States, with a further confirmatory trial required [19]. In colorectal cancer, single-agent activity was limited (ORR 9.7%), underscoring the need for combination approaches [20].

Real-world evidence indicates somewhat lower effectiveness of sotorasib than in pivotal trials, with real-world cohorts reporting objective response rates of approximately 30–32% and a median progression-free survival of approximately 5 months, reflecting broader patient heterogeneity, comorbidities, and disease complexity seen in routine practice [21]. Hepatotoxicity remained the most clinically significant real-world toxicity, with Grade ≥3 ALT/AST elevations in 28% of patients who had received anti-PD-L1 therapy within the preceding 12 weeks [21,22]. These findings emphasize careful patient selection, hepatic monitoring, and dose management to optimize outcomes with sotorasib [21].

### 3.2. Adagrasib

Adagrasib (MRTX849) is pharmacokinetically distinguished from sotorasib by a substantially longer half-life (~23 h vs. ~5 h), enabling twice-daily 600 mg dosing and resulting in more sustained plasma exposure consistent with prolonged covalent KRAS G12C inhibition [4,23]. Adagrasib exhibits clinically meaningful CNS activity in KRAS G12C-mutant NSCLC with untreated brain metastases (intracranial ORR ~42%, high disease control), likely reflecting extensive tissue distribution, CNS penetration with CSF concentrations above target IC_50_, and a pharmacokinetic profile, including modulation of P-glycoprotein efflux, that supports sustained intracranial exposure [24,25]. Adagrasib is a CYP3A4 substrate and a strong inhibitor of CYP3A4, a moderate inhibitor of CYP2C9 and CYP2D6, and a P-glycoprotein inhibitor, with QTc-prolonging potential, requiring careful medication reconciliation and ECG monitoring, particularly in patients with polypharmacy or cardiac comorbidities [26].

The phase I/II KRYSTAL-1 trial established adagrasib’s clinical efficacy in previously treated KRAS G12C-mutant NSCLC, demonstrating an ORR of 42.9%, median DoR of 8.5 months, median PFS of 6.5 months, and median OS of 12.6 months in 116 evaluable patients, with Grade ≥3 TRAEs in 44.8%, most commonly hepatotoxicity, diarrhea, nausea, and fatigue, supporting FDA accelerated approval in December 2022 [4]. In the KRYSTAL-1 intracranial cohort of patients with untreated KRAS G12C-mutant NSCLC and CNS metastases, adagrasib demonstrated a centrally reviewed intracranial ORR of ~42%, an intracranial disease control rate of ~90%, and a median intracranial duration of response of ~12.7 months [25]. In the phase III KRYSTAL-12 trial (*n* = 453), adagrasib demonstrated superiority over docetaxel in ORR (~32% vs. ~9%) and median PFS (~5.5 vs. ~3.8 months; HR 0.58; *p* < 0.001), while Grade ≥3 treatment-related adverse events occurred in ~47% of adagrasib-treated patients and ~46% of docetaxel-treated patients [27].

Adagrasib demonstrated meaningful CNS activity in patients with untreated KRAS G12C NSCLC brain metastases, achieving an intracranial ORR of 42% and a DCR of 90%, supporting a preference for adagrasib in patients with progressive brain metastases [25]. However, adagrasib’s more complex drug-drug interaction (DDI) profile, QTc-prolonging potential, and gastrointestinal toxicity contrast with sotorasib’s primarily hepatic toxicity and lower DDI burden [26]. STK11/KEAP1 co-mutations consistently predict reduced benefit with both agents (PFS 3–4 vs. 7–8 months in co-mutation-negative patients) [2,27]. Clinical selection should therefore consider CNS status, comorbidities, cardiac history, co-mutational landscape, and prior therapies to individualize therapy [26] (Table 1).

### 3.3. Emerging Next-Generation KRAS G12C Inhibitors

Preclinical in vitro mutagenesis studies have identified diverse secondary KRAS mutations that confer resistance to KRAS G12C inhibitors, with some (e.g., G13D, R68M, A59S/T) showing differential sensitivity to adagrasib versus sotorasib, suggesting that the pattern of secondary mutations might inform sequencing strategies [28]. The clinical success of sotorasib and adagrasib has catalyzed a rapidly expanding pipeline of next-generation KRAS-directed therapies that address the principal limitations of first-generation agents, including incomplete target occupancy, adaptive RAS-MAPK reactivation, limited mutation coverage, and inadequate response durability [29,30]. Among the most potent next-generation covalent G12C inhibitors, divarasib demonstrated an ORR of 53.4% in KRAS G12C-mutant NSCLC with a median DoR of approximately 14 months, representing a meaningful improvement in response depth and durability over first-generation agents [29]. Glecirasib has demonstrated promising clinical activity in KRAS G12C-mutant NSCLC, with objective response rates of approximately 48% and high target selectivity in preclinical studies [31], while early-phase data for olomorasib have shown preliminary intracranial activity in patients with active brain metastases [32]. Garsorasib (D-1553), a further covalent G12C(OFF) inhibitor, showed antitumor activity in KRAS G12C-mutant NSCLC in a phase I study [33] and has since received conditional NMPA approval in China, but not FDA or EMA approval, for previously treated disease (Table 2). Elisrasib (D3S-001), a distinct next-generation GDP-bound (OFF) G12C inhibitor, has reported early-phase objective response rates of approximately 57% in G12C inhibitor-naive previously treated NSCLC and meaningful activity (ORR ~32%) in G12C inhibitor-pretreated disease, including patients with CNS metastases [34,35]. Addressing the limitation of exclusive GDP-bound KRAS G12C inhibition, RMC-6291 is a tri-complex KRAS G12C(ON) inhibitor that targets the active GTP-bound conformation through cyclophilin A-mediated complex formation, with preclinical studies demonstrating potent suppression of KRAS signaling and activity in models resistant to KRAS G12C(OFF) inhibitors [36]. Beyond KRAS G12C, MRTX1133, a non-covalent, selective KRAS G12D inhibitor, has demonstrated potent inhibition of KRAS signaling and tumor regression in preclinical G12D-mutant pancreatic cancer models [10,36]. Broadening RAS inhibition beyond G12C, daraxonrasib (RMC-6236), an oral RAS(ON) multi-selective tri-complex inhibitor of the active GTP-bound state of mutant and wild-type RAS, has shown activity in previously treated RAS G12X-mutant NSCLC (objective response rate 38%, median progression-free survival 9.8 months, median overall survival 17.7 months) [37]. It is the most clinically advanced agent of this class: in the phase III RASolute 302 trial in previously treated metastatic pancreatic cancer, daraxonrasib improved median overall survival to 13.2 versus 6.7 months with chemotherapy (hazard ratio 0.40), the first positive randomized phase III result for a RAS(ON) inhibitor [38], and it is now being compared with docetaxel in previously treated RAS-mutant NSCLC in the phase III RASolve 301 trial [37] (Table 2).

## 4. Combination Strategies

### 4.1. KRAS G12C + EGFR Inhibition

KRAS G12C inhibition relieves negative feedback on upstream RTKs, particularly EGFR, triggering compensatory ligand-mediated signaling that restores ERK activity and limits single-agent efficacy [39]. This effect is most pronounced in colorectal cancer due to high basal EGFR dependency, providing a rationale for vertical co-inhibition [17]. In the KRYSTAL-1 phase 1/2 trial, adagrasib combined with cetuximab in heavily pretreated patients with metastatic KRAS G12C-mutant colorectal cancer achieved an investigator-assessed ORR of 46% with a DoR of 7.6 months and PFS of 6.9 months [16]. EGFR/RTK co-inhibition is being explored to overcome adaptive MAPK reactivation in KRAS G12C-mutant NSCLC, with CodeBreaK 101 demonstrating activity for sotorasib plus afatinib, and parallel evaluation of sotorasib plus panitumumab in colorectal cancer supporting vertical pathway inhibition [2,40].

### 4.2. KRAS G12C + SHP2 Inhibitors

SHP2 is a convergent signaling node through which multiple RTKs—including EGFR, MET, FGFR, and RET—reactivate RAS-MAPK signaling after KRAS G12C inhibition [18,41]. SHP2 blockade suppresses RTK-mediated adaptive resistance arising from diverse RTKs, supporting a broader strategy than targeting individual RTKs [18]. Preclinical studies show that the combination of the KRAS G12C inhibitor JDQ443 and the SHP2 inhibitor TNO155 yields enhanced tumor regression and delayed resistance compared with monotherapy in NSCLC models [42]. Early clinical data show manageable toxicity and preliminary efficacy, though prospective validation is needed before routine use [43].

### 4.3. KRAS G12C + SOS1 Inhibitors

SOS1 inhibitors target the guanine nucleotide exchange factor SOS1, preventing GDP-to-GTP RAS activation and thereby suppressing RAS signaling and RTK-mediated feedback reactivation [6]. BI 1701963, the first SOS1 inhibitor to enter clinical testing, has demonstrated preclinical synergy with MAPK pathway inhibitors and is being evaluated in early-phase trials in combination with KRAS G12C inhibitors [6,23]. Preliminary data indicate manageable safety and enhanced RAS suppression, though objective responses remain limited, emphasizing the importance of biomarker-driven patient selection [6,23,44].

### 4.4. KRAS G12C + Immunotherapy

KRAS G12C inhibition reverses oncogene-driven immunosuppression and promotes interferon-mediated remodeling of the tumor microenvironment, enhancing antigen presentation and supporting combination strategies with immune checkpoint blockade [45]. Clinical translation of sotorasib-immunotherapy combinations has been constrained by hepatotoxicity, with CodeBreaK 100/101 demonstrating Grade ≥3 hepatic adverse events in over 30% of patients [22]. Contributing factors include intrinsic G12C inhibitor hepatotoxicity, ICI-primed hepatic immunity, and short ICI-to-G12C inhibitor intervals; accordingly, current clinical experience supports ICI washout periods of 4–8 weeks prior to initiating KRAS G12C inhibitor therapy [22]. Adagrasib plus pembrolizumab in KRYSTAL-7 demonstrated a manageable safety profile with lower rates of severe hepatotoxicity, along with promising clinical activity, although randomized data confirming benefit over pembrolizumab alone are pending [5]. STK11 (LKB1) alterations in KRAS-mutant NSCLC are associated with an immunologically inert tumor microenvironment characterized by reduced T-cell infiltration and resistance to PD-1 blockade [11,12].

## 5. Mechanisms of Acquired Resistance

### 5.1. On-Target KRAS Mutations

Acquired resistance to covalent KRAS G12C inhibitors frequently arises through secondary on-target KRAS mutations that alter the switch-II binding pocket and impair inhibitor binding [28,46]. Comprehensive genomic analyses have identified a spectrum of secondary KRAS mutations, most notably Y96D, which directly disrupts the binding interface; H95 mutations (H95D, H95R, H95Q) that alter the switch-II pocket architecture; and R68S mutations that induce conformational changes [28,46,47]. These on-target resistance mutations frequently co-occur with other resistance alterations in a polyclonal pattern, and their differential distribution across sotorasib- and adagrasib-treated patients has informed the rationale for sequential KRAS G12C inhibitor use and the development of next-generation inhibitors, including RMC-6291, that target the active GTP-bound conformation [36,46,48].

### 5.2. Upstream Reactivation of RTK Signaling

Beyond on-target mutations, a dominant mechanism of acquired resistance involves upstream reactivation of RTK signaling that bypasses the need for functional KRAS G12C [46,49]. Post-progression genomic profiling has identified amplifications in multiple RTKs, including EGFR, HER2, FGFR1/2, MET, and ALK [46,49,50]. Central to this upstream reactivation is hyperactivation of SHP2, which functions as a convergent signaling node integrating inputs from multiple activated RTKs to drive GDP-to-GTP exchange on RAS; this provides the mechanistic basis for combining KRAS G12C inhibitors with SHP2 inhibitors [18,49]. Post-progression liquid or tissue biopsy profiling to identify the dominant RTK alteration driving bypass signaling is essential to inform rational subsequent therapy selection [46,49,50].

### 5.3. Downstream Signaling Reactivation

Resistance can also emerge through alterations entirely downstream of KRAS. Activating mutations in BRAF V600E and alterations in RAF/MEK signaling enable ERK reactivation that bypasses dependence on GTP-bound KRAS [46,50]. In parallel, activation of the PI3K-AKT-mTOR axis through PIK3CA mutations, PTEN loss, and AKT amplification provides an alternative survival signal [51], while YAP/TAZ transcriptional co-activator upregulation drives tumor cell survival through RAS-MAPK-independent mechanisms [52]. The convergence of MAPK rebound and parallel pathway activation underscores the necessity of vertical and horizontal co-targeting strategies [7,8,49].

### 5.4. Phenotypic Adaptations: EMT and Lineage Plasticity

**Figure 3:**
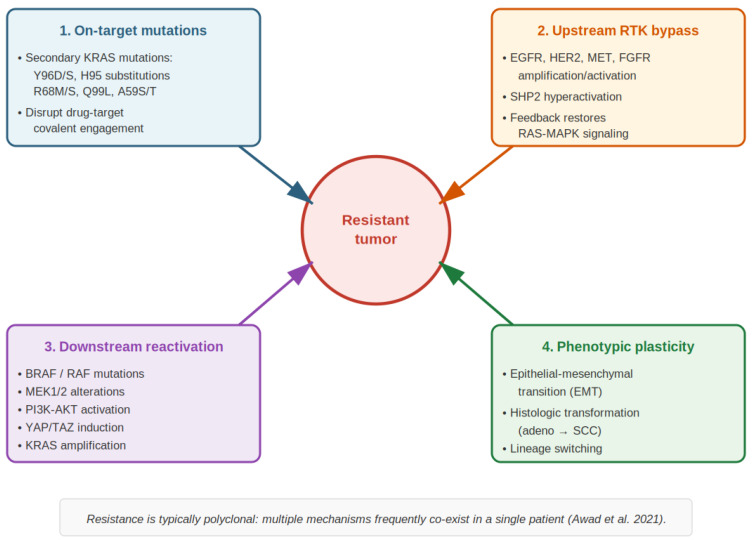
Mechanisms of acquired resistance to KRAS G12C inhibition. Resistance is organized into four main categories: (1) on-target secondary KRAS mutations altering the switch-II pocket (Y96D/S, H95, R68M); (2) upstream reactivation via RTK amplification (EGFR, HER2, MET, FGFR) and SHP2 hyperactivation; (3) downstream MAPK and PI3K pathway reactivation (BRAF, MEK, PI3K-AKT, YAP/TAZ); and (4) phenotypic plasticity including EMT and lineage switching. Resistance is typically polyclonal. Reference from [46].

Resistance can arise through non-genomic phenotypic adaptations that alter tumor cell identity and reduce dependence on KRAS signaling. Epithelial-to-mesenchymal transition (EMT), regulated by transcription factors including ZEB1 and SNAIL, has been implicated in resistance to KRAS G12C inhibitors [53,54]. Lineage plasticity, analogous to the EGFR-mutant NSCLC-to-SCLC transformation, occurs in a subset of KRAS G12C tumors, manifesting as neuroendocrine transdifferentiation, RB1/TP53 loss, and uncoupling from KRAS dependence [46,55,56]. These findings highlight the importance of integrating transcriptomic and histological assessments to guide therapy after progression [57] (Figure 3).

### 5.5. Role of Co-Mutations: STK11, KEAP1, and TP53

Co-occurring mutations in STK11/LKB1, KEAP1, and TP53 are key determinants of both intrinsic sensitivity and acquired resistance. STK11/LKB1 loss (~20–30% of KRAS-mutant lung adenocarcinomas) drives an immunologically cold tumor microenvironment, limiting efficacy of KRAS G12C inhibitor-immunotherapy combinations [58,59,60]. KEAP1 mutations (~15–20%) activate NRF2-dependent cytoprotective programs, correlating with inferior ORR, PFS, and OS, establishing KEAP1 as a negative predictive biomarker [12,25]. TP53 co-mutations drive genomic instability and clonal evolution, highlighting their relevance for biomarker-driven trial stratification [11,46].

## 6. KRAS G12C Therapy in Clinical Practice

### 6.1. Shifting Paradigms in Treatment Sequencing

The established role of sotorasib and adagrasib as second-line therapies is being increasingly challenged by emerging evidence suggesting that earlier deployment may yield superior outcomes in genomically defined patient subsets [12,61]. In patients harboring co-occurring STK11/LKB1 or KEAP1 mutations (~30–40% of KRAS G12C-mutant NSCLC), first-line platinum-based chemoimmunotherapy delivers substantially attenuated benefit, suggesting a compelling rationale for prioritizing KRAS G12C-directed therapy in the frontline setting [62,63,64]. Furthermore, prior immunotherapy exposure may augment subsequent KRAS G12C inhibitor hepatotoxicity through immune sensitization mechanisms, supporting a biomarker-driven sequencing framework that integrates STK11, KEAP1, and TP53 co-mutation status into frontline treatment allocation decisions [22,65].

### 6.2. First-Line Trials and Preliminary Data

Several prospective trials are evaluating KRAS G12C inhibitors in the frontline setting. KRYSTAL-7, evaluating adagrasib plus pembrolizumab versus pembrolizumab monotherapy in previously untreated PD-L1-high KRAS G12C-mutant NSCLC, has reported early signals of enhanced ORR, though Grade ≥3 hepatotoxicity rates of approximately 22% represent a clinically significant safety signal [5]. CodeBreaK 201, evaluating sotorasib plus pembrolizumab, similarly demonstrated concerning hepatotoxicity signals [66,67]. Regulatory pathways for frontline approval are anticipated to depend critically on mature PFS and OS data, with biomarker-enriched trial designs focusing on STK11/KEAP1 co-mutation status likely to define the approvable patient population [5,15].

### 6.3. CNS Disease Management

CNS metastases occur in approximately 30–40% of patients with advanced KRAS G12C-mutant NSCLC [68]. Adagrasib has demonstrated prospectively validated intracranial efficacy in the KRYSTAL-1 trial, with an intracranial ORR of 33–42% and an intracranial DCR of approximately 87–90% in patients with untreated brain metastases [25]. Sotorasib has no published prospective data supporting intracranial activity [68]. These data support adagrasib as the preferred agent in patients with CNS involvement, and emerging data suggesting meaningful intracranial activity with olomorasib further expand the CNS-active therapeutic toolkit [24,25,32].

### 6.4. Special Populations and Co-Mutational Landscapes

**Figure 4:**
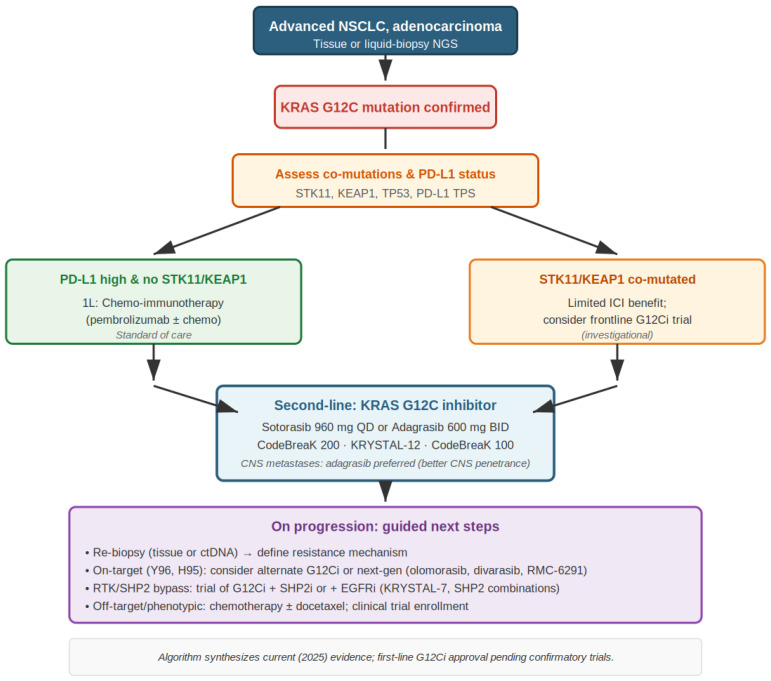
Proposed treatment algorithm for KRAS G12C-mutant advanced NSCLC. Following NGS confirmation of KRAS G12C, assessment of co-mutations (STK11, KEAP1, TP53) and PD-L1 status guides first-line therapy selection. Patients with PD-L1-high disease and no STK11/KEAP1 co-mutation receive chemoimmunotherapy; those with STK11/KEAP1 co-mutation are candidates for frontline G12C inhibitor trials (investigational). Second-line therapy consists of sotorasib or adagrasib (CNS metastases: adagrasib preferred). Post-progression management is guided by resistance biopsy. This algorithm represents the authors’ proposal, synthesized from the cited biomarker and trial evidence rather than from a single source or guideline. Outside of clinical trials, platinum-based chemoimmunotherapy remains the current standard first-line treatment for advanced KRAS G12C-mutant NSCLC, including in STK11/KEAP1-comutated disease; frontline KRAS G12C inhibition is investigational and not a routine standard-of-care option. KRAS G12C inhibitors (sotorasib, adagrasib) are approved only in the second-line or later setting.

KRAS G12C-mutant NSCLC disproportionately occurs in current or former smokers, in whom high tumor mutational burden may theoretically enhance immunotherapy sensitivity; however, the frequent co-occurrence of STK11 and KEAP1 mutations substantially attenuates immunotherapy benefit, rendering genomic co-mutation profiling more clinically informative than smoking history alone [69,70]. Never-smokers harboring KRAS G12C represent a biologically distinct minority with lower TMB, further supporting KRAS-directed therapy prioritization [69]. Among co-mutational subsets, STK11-mutant tumors demonstrate the most profound immunotherapy resistance; KEAP1-mutant tumors exhibit reduced KRAS inhibitor sensitivity requiring NRF2 pathway co-targeting; and TP53 co-mutations may warrant earlier integration of combination approaches [13,64] (Figure 4).

### 6.5. Real-World Effectiveness, Access, and Quality of Life

Beyond controlled trial populations, real-world effectiveness has been more modest than pivotal-trial estimates: registry and single-institution cohorts report lower objective response rates and shorter progression-free survival for sotorasib than in CodeBreaK 100/200, reflecting the broader comorbidity, poorer performance status, and higher burden of CNS involvement seen in unselected patients [42]. Patient-reported and quality-of-life outcomes remain comparatively under-reported. CodeBreaK 200 documented delayed deterioration in global health status and physical functioning with sotorasib relative to docetaxel, but adagrasib and most next-generation agents still lack mature quality-of-life data, and gastrointestinal and hepatic toxicities meaningfully affect tolerability and dose intensity in practice. Systematic incorporation of patient-reported outcomes into frontline and combination trials is needed to establish whether biochemical and radiographic gains translate into patient-centered benefit.

Cost and access considerations further shape real-world use. As orally administered targeted agents priced at specialty-drug levels, KRAS G12C inhibitors carry substantial financial toxicity for patients and payers, and access is constrained in health systems lacking reflex next-generation sequencing or reimbursement for comprehensive genomic profiling. Because KRAS G12C status is identified predominantly through broad NGS panels rather than single-gene testing, the cost-effectiveness of frontline KRAS-directed therapy is inseparable from the cost and availability of molecular diagnostics. These practical determinants—diagnostic access, drug cost, and toxicity-related discontinuation—are likely to influence population-level outcomes as much as incremental efficacy gains, and warrant explicit evaluation alongside clinical endpoints.

## 7. Discussion

The emergence of KRAS G12C as a tractable therapeutic target exemplifies the convergence of structural biology, medicinal chemistry, and clinical oncology in redefining a disease once considered biochemically impervious. The cryptic switch-II pocket, the nucleophilic reactivity of the mutant cysteine, and the preserved nucleotide cycling of KRAS G12C collectively enabled a mutant-selective covalent strategy [1,2,3,4]. This explains why G12C inhibitors have outpaced earlier RAS-directed modalities—including farnesyl transferase inhibitors, MEK inhibitors as monotherapy, and RAS vaccines [29,36,48].

Yet the same biology that confers susceptibility also constrains durability. Response depth in pivotal trials was moderate (ORR 28–43%), median PFS rarely exceeded seven months, and OS benefit in CodeBreaK 200 was not statistically demonstrable [2,4,19,27]. These limitations reflect incomplete target occupancy, feedback reactivation through EGFR and other RTKs, and the heterogeneous co-mutational landscape [11,12,15,39,62]. Real-world cohorts consistently report lower effectiveness, underscoring that patient selection must account for comorbidities, prior immunotherapy exposure, and hepatic reserve [21,22].

The accumulating evidence supports a biologically stratified repositioning toward earlier treatment lines. Chemoimmunotherapy performs poorly in STK11- and KEAP1-comutated disease, and prior immunotherapy exposure increases hepatic toxicity risk [22,62,63,64,65]. However, current first-generation agents may lack the ORR and PFS/OS magnitude required for frontline positioning absent effective combination partners; compared with EGFR/ALK inhibitors, KRAS G12C therapies yield shorter disease control, reflecting greater pathway redundancy. Next-generation inhibitors with improved potency, CNS activity, and RAS(ON) coverage, combined with rationally designed vertical and immune combinations, are positioned to consolidate this shift [5,6,15,18,29,32,41,42].

### Strengths and Limitations

Several limitations qualify the conclusions of this review. First, it is a narrative rather than a systematic synthesis; studies were selected by author judgment, no formal risk-of-bias appraisal was undertaken, and relevant work may have been omitted. Second, much of the evidence supporting a move toward earlier treatment lines derives from early-phase, single-arm, or interim analyses and from conference abstracts not yet subject to full peer review, which are susceptible to publication and reporting bias and to attrition of early efficacy signals in mature data. Third, several comparisons between agents—most notably sotorasib versus adagrasib—rest on cross-trial comparisons of separate studies with differing eligibility criteria, endpoints, and follow-up; these are descriptive and hypothesis-generating rather than definitive, and no head-to-head randomized data exist. Fourth, the field is evolving rapidly, so regulatory status, trial readouts, and combination data cited here may be superseded. These constraints warrant cautious interpretation, particularly of statements regarding first-line positioning, which remains investigational pending randomized phase III confirmation.

## 8. Future Directions

Drug development is rapidly evolving beyond first-generation G12C(OFF) covalent inhibitors. Next-generation agents aim to deepen responses through enhanced target occupancy, more favorable pharmacokinetics, and improved CNS penetration, with early-phase data showing response rates approaching 50% [29,31,32]. Tri-complex RAS(ON) inhibitors target the active GTP-bound conformation and may circumvent switch-II pocket-dependent resistance [36]. Allele-selective non-covalent inhibitors of KRAS G12D and emerging pan-KRAS degraders further widen the therapeutic aperture [10,48]. Agents with improved toxicity profiles are also needed to consider adjuvant therapy or consolidation strategies.

Combinatorial design should prioritize mechanism-informed pairings. Vertical pathway inhibition addresses adaptive feedback and has shown clinical signal in colorectal cancer [16,17,18,40,41,42]. SOS1 inhibition offers a complementary node [6,44]. YAP/TAZ-TEAD inhibition represents a promising orthogonal strategy for tumors with transcriptional bypass resistance [52].

Biomarker-driven personalization will be central. ctDNA-based early response assessment offers a dynamic decision tool to escalate, maintain, or switch therapy in real time [15]. Co-mutation profiling (STK11, KEAP1, TP53, SMARCA4) should guide frontline allocation [62,63,64,70]. Additional emerging biomarkers including ctDNA burden, TMB, and CDKN2A/B loss may further refine patient selection [15]. Future trials should embed biomarker enrichment, serial ctDNA monitoring, and adaptive randomization [5,15,66].

## 9. Conclusions

KRAS G12C has traveled farther in a decade than any oncogenic driver previously labeled undruggable. Covalent inhibitors have moved from proof-of-concept to regulatory approval (accelerated approval in NSCLC in the United States), with sotorasib and adagrasib now established second-line standards supported by phase III superiority over docetaxel in PFS [2,4,19,27]. Emerging evidence increasingly supports a shift toward first-line KRAS G12C inhibition in genomically defined subgroups—particularly STK11- and KEAP1-comutated tumors—where chemoimmunotherapy underperforms [62,63,64]. First-line KRAS G12C inhibition nonetheless remains investigational: platinum-based chemoimmunotherapy is the current standard first-line option outside of clinical trials, and a frontline indication will require confirmation from randomized phase III trials. Durable benefit remains constrained by on-target switch-II pocket mutations, upstream RTK and SHP2-mediated bypass signaling, downstream MAPK and PI3K-AKT reactivation, phenotypic plasticity, and the co-mutational landscape [13,28,46,49,52]. Continued progress will depend on next-generation inhibitors covering both KRAS conformations and additional RAS alleles, mechanism-informed combinations, and biomarker-enriched trial designs [15,29,32,36]. The field’s central task in the coming years is to translate mechanistic clarity into durable survival benefit for the patient populations most likely to benefit.

## Figures and Tables

**Table 1:** Cross-trial comparison of sotorasib and adagrasib in previously treated KRAS G12C-mutant NSCLC. No randomized head-to-head trial of sotorasib versus adagrasib has been conducted; values are drawn from separate trials with differing designs, eligibility criteria, and populations and are therefore descriptive and hypothesis-generating rather than directly comparative.

Adagrasib (MRTX849)	Sotorasib (AMG 510)	Feature
Irreversible covalent binding to Cys-12 in GDP-bound state	Irreversible covalent binding to Cys-12 in GDP-bound state	Mechanism
600 mg orally twice daily	960 mg orally once daily	Dose
~23 h	~5 h	Half-life
KRYSTAL-1 (Ph I/II), KRYSTAL-12 (Ph III)	CodeBreaK 100 (Ph I/II), CodeBreaK 200 (Ph III)	Pivotal trials
42.9% (Ph II); 32% (Ph III)	37.1% (Ph II); 28.1% (Ph III)	ORR (pretreated)
6.5 mo (Ph II); 5.5 vs. 3.8 mo (Ph III)	6.8 mo (Ph II); 5.6 vs. 4.5 mo (Ph III)	Median PFS
12.6 mo (Ph II); OS pending	12.5 mo (Ph II); no OS benefit over docetaxel	Median OS
IC ORR ~42%, DCR ~90%	No prospective data	CNS activity
Nausea, diarrhea, hepatotoxicity, QTc prolongation	Hepatotoxicity (ALT/AST), diarrhea	Principal toxicities
Strong CYP3A4 inhibitor/substrate; P-gp inhibitor	Lower DDI burden; PPIs reduce absorption	DDI profile
Accelerated December 2022	Accelerated approval May 2021 (NSCLC); regular approval declined (FDA Complete Response Letter, Dec 2023)—remains accelerated approval	FDA status

ORR, objective response rate; PFS, progression-free survival; OS, overall survival; CNS, central nervous system; DDI, drug–drug interaction.

**Table 2:** Next-generation KRAS-directed agents and rational combination partners in clinical development (2024–2026).

Key Trial(s)	Stage	Mechanism	Class/Target	Agent
NCT04449874	Ph I/II	Next-gen covalent, higher selectivity/potency	KRAS G12C (OFF)	Divarasib (GDC-6036)
NCT05009329	Ph II	Covalent; low GI toxicity; NMPA-approved (China, May 2025) for ≥2 L NSCLC; not FDA/EMA-approved	KRAS G12C (OFF)	Glecirasib (JAB-21822)
LOXO-435 study	Ph I/II	2nd-gen covalent with improved selectivity	KRAS G12C (OFF)	Olomorasib (LY3537982)
NCT04585035	Ph II	Covalent; conditionally NMPA-approved (China, Nov 2024) for ≥2 L NSCLC; not FDA/EMA-approved	KRAS G12C (OFF)	Garsorasib (D-1553)
NCT05462717	Ph I	Tri-complex inhibitor targeting GTP-bound state	KRAS G12C (ON)	Elironrasib (RMC-6291)
NCT05379985; RASolute 302	Ph I/III	Multi-selective tri-complex inhibitor	Pan-RAS (ON)	Daraxonrasib (RMC-6236)
Preclinical → early clinical	Ph I	Non-covalent selective G12D inhibitor	KRAS G12D	MRTX1133
NCT04111458	Ph I	Blocks SOS1-KRAS interaction	SOS1 inhibitor	BI-3406/BI 1701963
Multiple combination trials	Ph I/II	Blocks RTK → RAS feedback	SHP2 inhibitor	TNO155/RMC-4630

G12C (OFF), inactive GDP-bound state inhibitor; G12C (ON), active GTP-bound state inhibitor; Pan-RAS (ON), multi-selective inhibitor.

## Data Availability

No new data were created or analyzed in this study. Data sharing is not applicable to this article.

## Abbreviations

The following abbreviations are used in this manuscript: ctDNA, circulating tumor DNA; DCR, disease control rate; DoR, duration of response; EGFR, epidermal growth factor receptor; EMT, epithelial-to-mesenchymal transition; FDA, U.S. Food and Drug Administration; GAP, GTPase-activating protein; GEF, guanine nucleotide exchange factor; HR, hazard ratio; ICI, immune checkpoint inhibitor; KEAP1, Kelch-like ECH-associated protein 1; KRAS, Kirsten rat sarcoma viral oncogene homolog; MAPK, mitogen-activated protein kinase; NGS, next-generation sequencing; NSCLC, non-small cell lung cancer; ORR, objective response rate; OS, overall survival; PD-1/PD-L1, programmed cell death protein 1/programmed death-ligand 1; PFS, progression-free survival; RTK, receptor tyrosine kinase; S-IIP, switch-II pocket; SHP2, Src homology region 2-containing protein tyrosine phosphatase 2; SOS1, son of sevenless homolog 1; STK11, serine/threonine kinase 11; TMB, tumor mutational burden; TRAE, treatment-related adverse event.
